# The role of confocal microscopy in the dermato–oncology practice

**Published:** 2011-02-25

**Authors:** A Diaconeasa, D Boda, M Neagu, C Constantin, C Căruntu, L Vlădău, D Guţu

**Affiliations:** * Department of Dermatology, ‘Gr. Alexandrescu’ Children's Hospital, BucharestRomania; ** ‘Carol Davila’ University of Medicine and Pharmacy, BucharestRomania; *** ‘Victor Babes’ National Institute of Pathology, BucharestRomania; **** Department of Pathology, ‘N. Paulescu’ National Institute of Diabetes, Nutrition and Metabolic Diseases, Bucharest Romania

**Keywords:** optical biopsy, dermatoscopy

## Abstract

Reflectance–mode confocal microscopy (RCM) is a new in vivo skin imaging technique. We present our one–year experience in RCM examinations in skin tumors and the retrospective analysis of patients enrolled in the Dermatological Department of ‘N. Paulescu’ Institute using the Fotofinder Dermoscope IIŴ for the dermatoscopy analysis and VivaScope 1500Ŵ  for in vivo RCM. We established the rank of RCM in the complex algorithm of skin cancer diagnose, showing that the presented experience can open new possibilities to implement this automated image analyzing system in the routine practice. Our analyzed cases clearly showed that confocal microscopy, therefore, optical biopsy, could guide the clinician towards an accurate diagnosis before surgical removal. Moreover, we emphasized that the development of this technique increases the potential of future teledermatologic applications.

## Introduction

Reflectance–mode confocal microscopy (RCM) is a new in vivo skin imaging technique. The resolution of emerging images is similar to that of classic microscopy (approx. 1ॖm), with a penetration depth of maximum 200 ॖm. This method allows observation of the cutaneous micromorphology in vivo, in real time, therefore realizing the so–called optical biopsy. 

The principle of in vivo confocal laser scanning microscopy consists of a coherent light of a laser that emits near infrared (700/2500 nm), projected through a lens system on a skin area. The reflected light is caught through an objective and then analyzed. The images obtained through this method have a similar resolution to that of classic microscopy, but they are black and white, horizontal, and parallel with the surface of the skin [1, 2]. In the reflectance mode, the skin imaging is based on different reflection indices of the micro anatomical structures and individual cells. This gives the image its natural contrast. The varying reflection of the laser light is collected through a small aperture and optically conjugates on focal planes (confocal planes), which are analyzed and consecutively transformed into a digital image with different levels of gray. Melanin offers the strongest contrast (reflection index = 1.7); therefore, the cytoplasm of melanocytes appears as intensely white. Keratin reflects less intensely (reflection index = 1.5), so that the cytoplasm of keratinocytes appears darker. Cell nuclei also appear dark and collagen appears very bright [3].

This skin imaging technique represents a non–invasive, less painful and non–destructive tissue method. The skin is unaffected during preparing procedures, thus minimizing the artifacts. The data collected in real–time are rapidly acquisitioned and processed and the segment of analyzed skin could be re–examined in order to evaluate the dynamic changes, such as the response to therapy. The numerous and still increasing applications of this method are currently considered an important research topic. 

The present paper subscribes to the recent clinical perspective, which states that the study of skin cancers turns into an established, highly specialized discipline, namely dermato–oncology. The steadily increasing skin cancers' incidence, as the most frequent type of tumor, and the morbidity dynamics represent a clinical challenge, because they are often sub–diagnosed by the clinician or ignored by the patient. The non–melanoma skin cancers (typically basal cell carcinoma and squamous cell carcinoma) are the most frequent categories of cancer in the Caucasian population, representing over 30% of the adult cancers in the USA [4]. In the last four decades, their incidence has regularly been increasing throughout the world with an annual rate of 3 to 8%, due to the actinic aggression, constant exposure to carcinogens, geographic changes, sun exposure and, last but not least, the skin photo–type [5]. The incidence of non-melanoma skin cancers in Europe is estimated to be of 0.12% [4]. Moreover, a patient with a non-melanocytic skin cancer can subsequently develop a higher probability of other types of cutaneous cancers in the next few years. Thus, 36.39% of the patients develop similar tumor lesions during the first year, after the initial diagnosis [6]. Among other oncological disorders, the malignant melanoma (MM) is one of the most aggressive types of tumor, having a continuously growing incidence, a high morbidity and mortality, affecting the young population, with substantial costs for treatment.  In the case of cutaneous tumors, the most adequate clinical approach is screening and early diagnosis. The recent development of imaging techniques offers the opportunity for in vivo assessment of the skin, in a non–invasive and high–resolution way, thus improving the disadvantages of biopsy and histopathology analysis, known to be painful, time–consuming and very expensive with regard to procedures. Dermatoscopy and, recently, the reflectance–mode confocal microscopy are of practical interest in the detection of cutaneous cancers.

The dermatoscopy has already become a routine procedure, while the RCM applications in dermato–oncology represent an imporatant research area, including improvement of diagnostic accuracy, improved assessment of dermatoscopic–histologic correlation, in vivo biopsy site selection, response control of conservative therapies and surgical margin assessment (including Mohs' micrographic surgery) [7,8].

**Figure 1:**
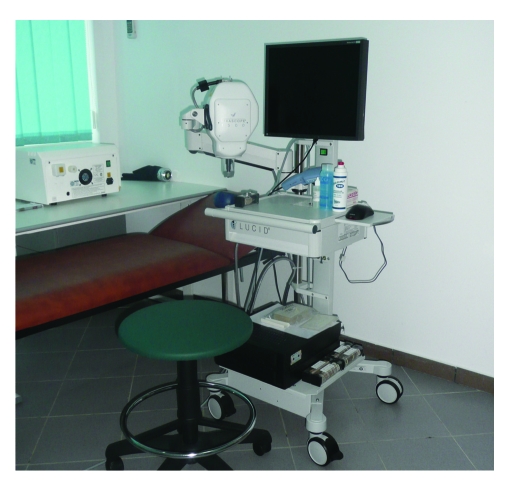
Reflectance–mode confocal microscopy VivaScope 1500

Recently, we have implemented the VivaScopeŴ 1500 equipment (Lucid Inc, Rochester, NY) (Figure 1), which comprises an individual laser combination (830 nm, 785 nm and 642 nm) and includes the fluorescent specific filters (LUCID) of 642 and 785 nm. The major characteristic of this hybrid is the possibility to visualize the tissue images both in reflectance and fluorescence mode (by simply adding the filter), to about 200 ॖ in depth (papillary or profound derma).

This paper reflects our one–year experience in RCM examinations in skin tumors and sums up our conclusions regarding the evaluation and implementation of in vivo confocal laser scanning microscopy in clinical practice. More specifically, the aim of the presented study was the retrospective analysis of the patients enrolled in the Dermatological Department of ‘N. Paulescu’ Institute using the Fotofinder Dermoscope II for the dermatoscopy analysis and VivaScope 1500 for in vivo reflectance–mode confocal microscopy, both equipments were purchased by a research grant (*26/CP/I/2007*) to establish the Excellence Research Center of UMF ‘Carol Davila’.

## Materials and methods

The results obtained were analyzed using several selection criteria for the investigated cases.

The first selection criterion was the examination of cutaneous tumor lesion in patients registered from September 15, 2009 to September 15, 2010. The second phase of the selection implies the following inclusion criteria: the existence of captured clinical and dermatoscopy images for the examined lesion; the existence of recorded images for reflectance–mode confocal microscopy examination; the complete surgical excision followed by histo–pathological evaluation, which also confirmed the final diagnostic. 

Teams of two experienced physicians were completed in order to perform the analysis of clinical, dermatoscopic and confocal microscopy images. For every type of examination, a presumably diagnostic was emitted by a consensus between those two clinicians. Accordingly, even two or none presumably diagnostic hypotheses were admitted. Finally, all three diagnostic hypotheses (clinical, dermatoscopical and confocal microscopy) were recorded in a table and compared with the final certifying histopathological diagnostic.  The working data were completed as appropriate with an observation box related to anamnesis or additional investigations, which lead to the excision and therapeutical decision. 

The interpretation of dermatoscopic images, in order to give out a presumably diagnostic based on this investigation, was performed on ‘Pattern Analysis‘, ‘ABCD Rule’ and ‘7–Point Checklist’ diagnosis algorithms [9].

The presumably diagnostic of the recorded images upon examination with VivaScope 1500 equipment, was done by two physicians with over one year expertise in the interpretation of this methodology (trained in the Department of Dermatology, University of Modena and Reggio Emilia, tutor Prof. dr. Giovanni Pellacani). The physicians examined the cases based on their up–to–date knowledge regarding the multitude of diagnostic criteria for in vivo RCM in skin tumors [10–18]. 

After applying the selection criteria, a number of 38 tumor lesions were subsequently investigated; their certitude diagnosis has been confirmed by histopathological analysis and it is summarized in Table 1. The patients' group had a medium age of 50 and the male/female ratio (m/f) was 18/20.

**Table 1:** Studied tumors, male–to–female ratio and median age in accordance with specific pathology

Tumor	Number	m/f	Median age
Nevus	4	1/3	38
Atypical/dysplastic nevus	12	5/7	36
Basal cell carcinoma	9	5/4	62
Actinic keratosis	3	2/1	65
Squamous cell carcinoma	1	1/–	68
Melanoma	7	3/4	55
Hemangioma	1	–/1	21
Other	1	1/–	55
Total	38	18/20	50

## Results

Five clinical situations were identified following the examination of clinical, dermatoscopical and confocal microscopy images and the further correlation with certitude histopathological diagnostic has been established.

**Figure 2:**
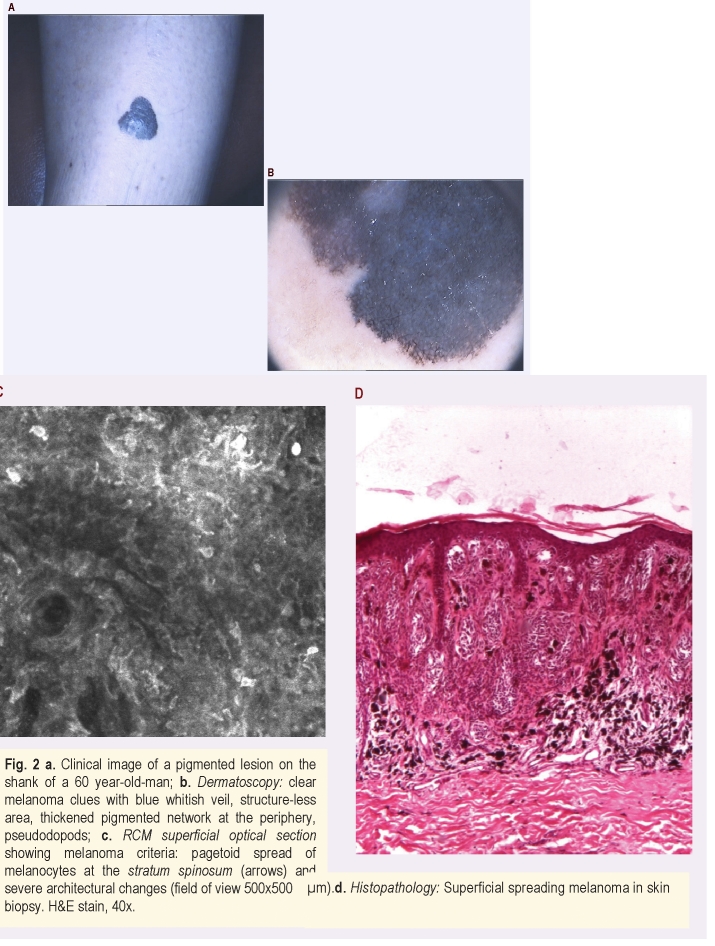
Clinical image of a pigmented lesion on the shank of a 60 year–old–man; b. Dermatoscopy: clear melanoma clues with blue whitish veil, structure–less area, thickened pigmented network at the periphery, pseudodopods; c. RCM superficial optical section showing melanoma criteria: pagetoid spread of melanocytes at the stratum spinosum (arrows) and severe architectural changes (field of view 500x500 ॖm).d. Histopathology: Superficial spreading melanoma in skin biopsy. H and E stain, 40x.

**Figure 3:**
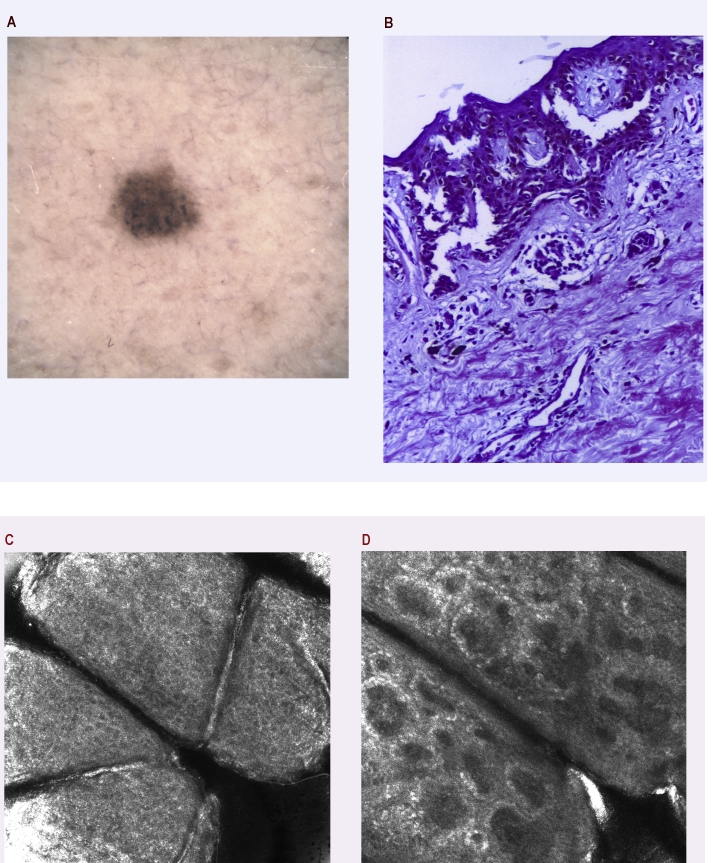
a. Dermatoscopy of a pigmented lesion in a patient with a history of melanoma: few irregular dots and globules corresponding to melanocytic nests seen on pathology, no clues for melanoma though; b. Histopathology: Junctional dysplastic nevus with architectural and cytological atypia; there is a mild superficial perivascular lymphocytic infiltration. H and E stain, 100x. c. RCM superficial optical section showing regularly arranged uniform keratinocytes in a honeycomb pattern in stratum granulosum (500x500 ॖm). d. RCM deeper optical section with edged papillae at dermal–epidermal junction; RCM images suggest a benign lesion.

The first situation points out a definite presumably clinical diagnostic, positively correlated with dermatoscopical and confocal microscopy examination and also with the histopathological diagnostic. In this topic, 3 cases of nevi, 3 cases of atypical nevi, 4 cases of basal cell carcinoma, one case of actinic keratosis, 5 cases of melanoma, namely, a total of 16 cases for the first situation are included. In this case, the imagistic exploration is correlated with the histopathological diagnosis. We illustrate this clinical condition in two cases, respectively one melanoma case (Figure 2) and one nevus, in a patient with previous circumstances of melanoma (Figure 3), who was periodically monitored for newly emerged pigmentary lesions.

The second situation is represented by the lack of a clinical diagnostic and the existence of a dermatoscopic one, augmented by confocal microscopy and confirmed by histopathological analysis. Accordingly, 7 cases of atypical nevi were included, 4 cases of basal cell carcinoma, one case of actinic keratosis and 2 cases of melanoma, a total of 14 cases for this condition. This is an obvious example regarding the benefit of imagistic explorations (dermatoscopy and confocal microscopy) for the clinicians and for pre–surgery diagnosis accuracy, which was therefore greatly enhanced in dermato–oncology. 

**Figure 4:**
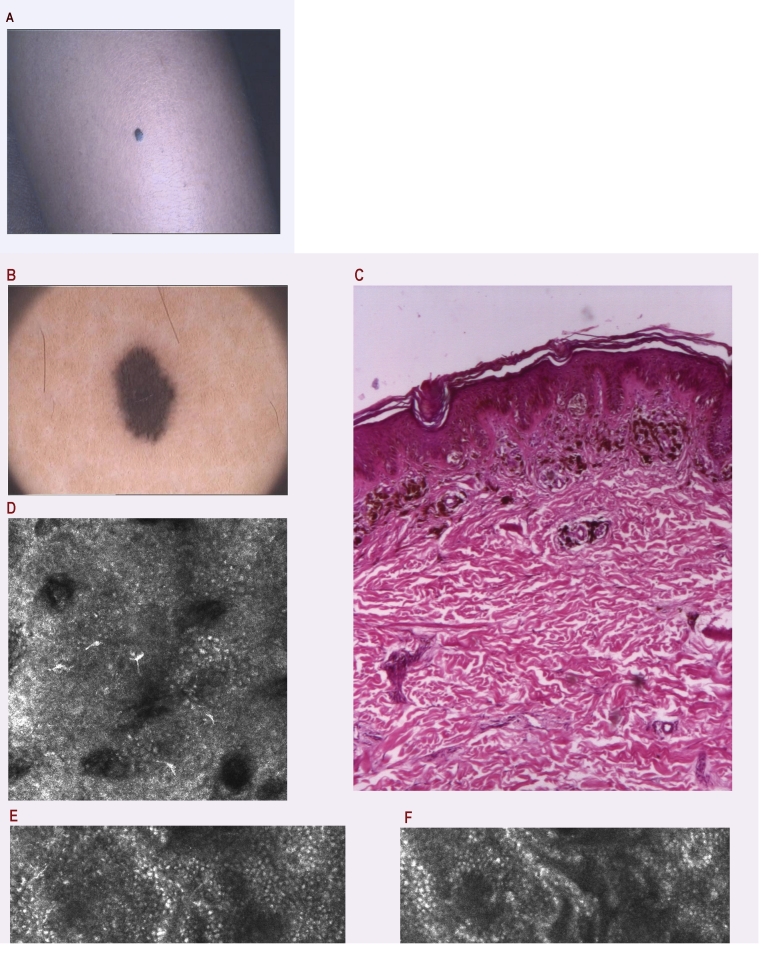
a. Clinical aspect of a recently appeared pigmented lesion on the calf of the leg of a 15–year–old girl; b. Dermatoscopy: structureless area, few peripheric structures; possible Reed nevus with heavily pigmented cells, Clark nevus with compactly aggregated nest of melanocytes; blue nevus or melanoma cannot be excluded; c. Histopathology: Lentiginous junctional nevus; single cells and nests of nevus cells are arranged at the tips of rete ridges; the upper dermis contains an infiltration of melanophages and mononuclear cells. H and E stain, 40x. d. RCM superficial optical section showing few pagetoid cells (arrows); e. RCM deeper optical section with normal stratum basale; f. RCM deep optical section with edged papillae (arrows) and normal melanocytes nests, suggesting a nevus.

**Figure 5:**
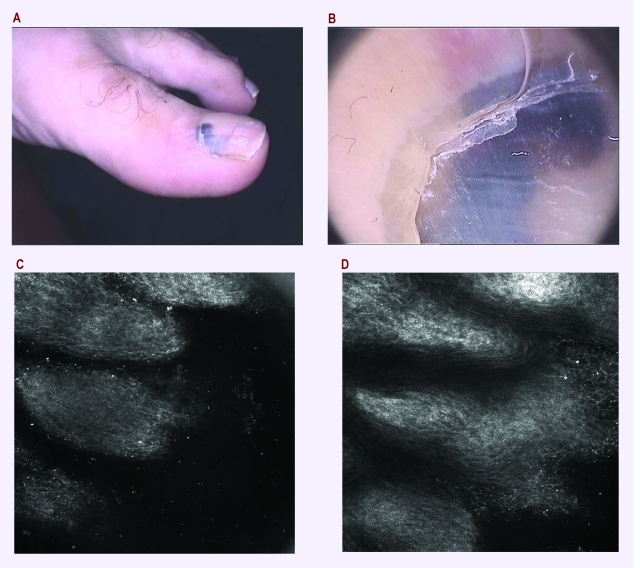
a. Clinical aspect of a nail lesion in a 55–year–old man; b. Dermatoscopy: pseudo–Hutchinson sign, no clear clues for melanoma–possible hemorrhage; c. RCM superficial optical section of the nail fold with normal stratum corneum and no atypical cells (500x500 microm); d. Deeper RCM image showing no sign of melanoma, suggesting a subungual hemorrhage (as confirmed after surgery).

**Figure 6:**
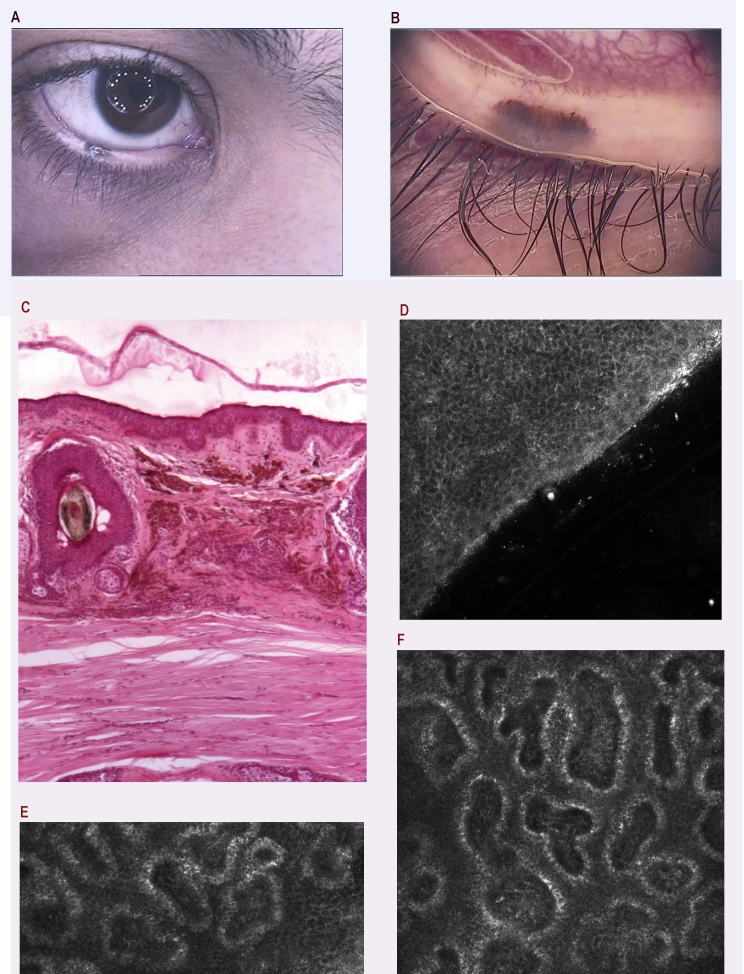
a. Pigmented lesion of the eyelid in a 17–year–old boy; b. Dermatoscopy: unspecific dermatoscopic pattern due to the particular location; if we are following the classical  pattern analysis we can differentiate 2 colors, irregularly distributed, few dots in the periphery and also some peripheral linear structures, so it is impossible to exclude a melanoma after this assessment; c. Histopathology: Intradermal nevus; the upper dermis contains nests of nevus cells and a moderate amount of melanin. H and E stain, 40x. d. RCM superficial optical section showing a normal stratum spinosum (500x500 µm); e. Deeper RCM image with edged papillae (as a sign of a benign lesion); f. Typical RCM aspect of a nevus with melanin providing strong cytoplasmic contrast at dermal–epidermal junction.

The third condition refers to the absence of an evident outcome upon clinical and dermatoscopic examinations, although confocal microscopy points out the correct diagnostic even before the surgical excision. There are 3 such cases in our study, presented in this section. The first case (Figure 4) is a girl, aged 15, with pigmentary lesions, who raised the suspicion of melanoma after dermatoscopy investigation. The confocal microscopy exploration sustained the benign nature of the lesion. The second case (Figure 5) was a pigmentary lesion of nail fold, suspected of acral melanoma. The RCM examination revealed the benign aspect at the nail fold level and indicated the subungual hemorrhagic diagnosis.  The third case (Figure 6) was a young man with a pigmentary eyelid lesion recently emerged. Moreover, the melanoma diagnosis was under suspicion. The confocal microscopy does not confirm the melanoma suspicion, none of the melanoma criteria being present. Consequently, the surgical excision for diagnosis purposes was made with the smallest safety border and thus, with post–surgical minimum defect.

**Figure 7:**
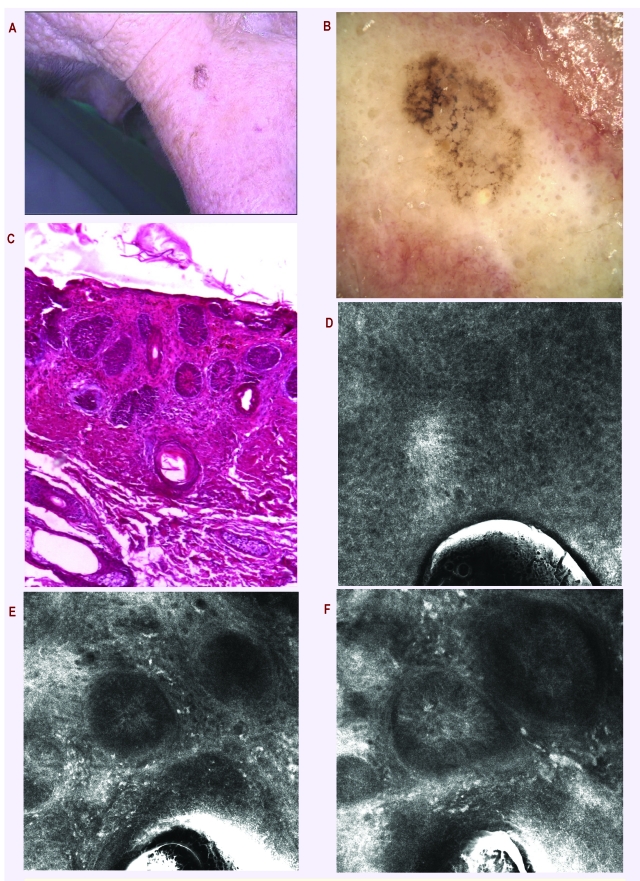
a. Pigmented lesion on the face of a women aged 65; b. Dermatoscopy: very suspicious lesion for melanoma with annular granular pattern, dots invading the follicular openings, pseudo pigmented thickened network; no clue for BCC despite the clinical aspect, no arborising vessels, nor blue gray ovoid nests; possible lentigo maligna melanoma, pigmented actinic keratosis or lichen planus–like actinic keratosis; c. Histopathology: Nodular basal cell carcinoma; nestsof tumor cells show peripheral palisading. H and E stain, 40x. d. RCM superficial optical section showing a normal stratum spinosum (500x500 micro m); e. Deeper RCM optical section revealing nodular type basal cell carcinoma with palisading and clefting (arrow); f. RCM image showing inflammatory infiltrates and leukocyte adhesion and rolling (arrows); RCM suggests a diagnosis of basal cell carcinoma.

The forth situation is represented by a basal cell carcinoma (Figure 7), where confocal microscopy points out the correct diagnostic, and, totally excludes the previous clinical and dermatoscopical suspicion. We can highlight the complementary character of this investigation through this case. 

The fifth situation has been exemplified by a single case, where we noticed an inconsistency between clinical, dermatoscopical and ultrasonography assessment (which raised the suspicion of solid malignant tumor). The confocal microscopy was crucial in this case, with respect to the vascular lesion diagnosis, finally confirmed after the surgical excision. 

**Figure 8:**
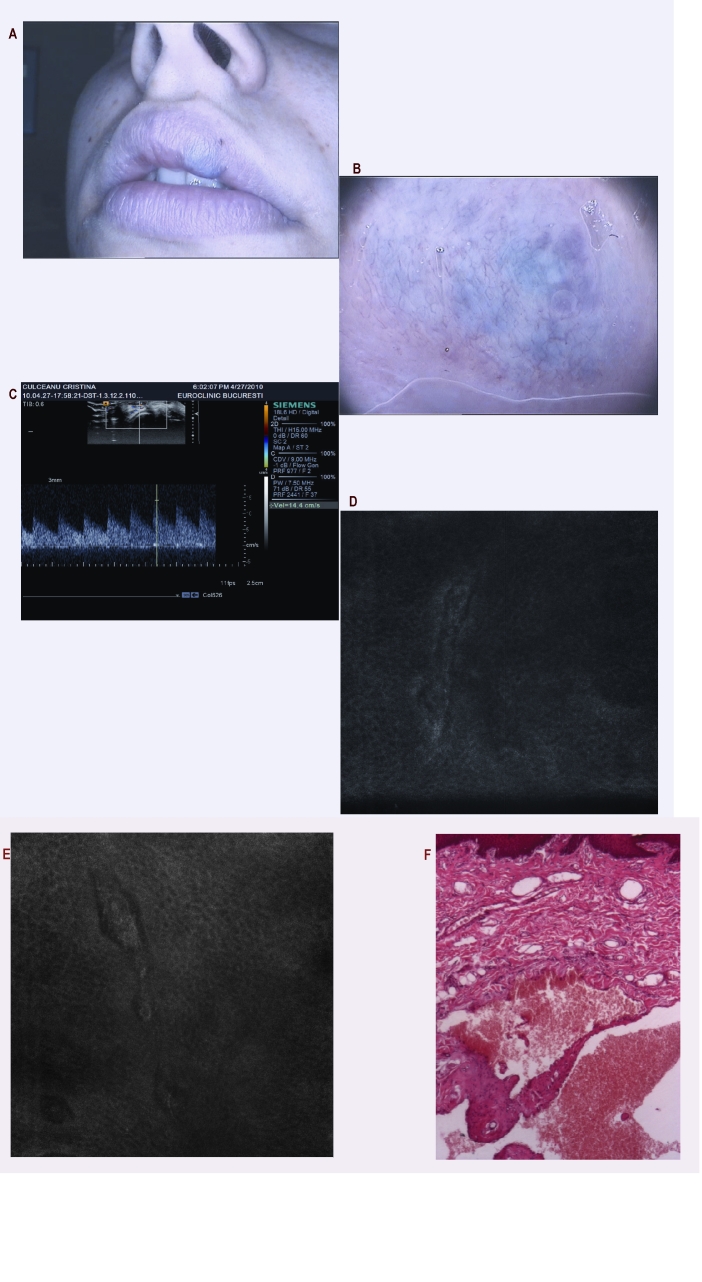
a. Clinical aspect of a blue tumor on the upper lip in a women aged 21;  b. Dermatoscopy: red and blue lagoons–specific pattern for a vascular structure; c. Ultrasonography examination suggesting a solid tumor; after this examination the surgical excision is decided; d and e. RCM images showing capillary loops and still suggesting a vascular lesion (500x500 micro micro m field of view); f. Histopathology: Dilated vascular spaces filled with erythrocytes and lined by a single layer of flattened endothelial cells and a thin wall of fibrous tissue. H and E stain, 40x.

Finally, there was a number of 3 cases (one case of atypical nevi, one of actinic keratosis and one case of squamous cell carcinoma) in our study which could not be correctly diagnosed previously to the surgical excision

## Discussions

Nowadays, the diagnosis of skin tumors in the daily practice involves the following steps: clinical examination, dermatoscopy, other imaging methods (i.e. ultrasonography). Reflectance–mode confocal microscopy has recently started to be integrated in the daily practice of dermato–oncology departments. Even though the sensibility and specificity of all these methods are very high, dermato–pathology is the final and essential step that shows the exact diagnosis of a skin tumor. 

Regarding the accuracy of pigmentary tumor lesion diagnosis, recent studies based on data analysis of over 50,000 lesions have demonstrated that this method possesses a greater sensitivity than that of dermatoscopy [19], but a similar specificity [20]. All these data sustain that these two methods are complementary. Currently, there have been several attempts to characterize some diagnosis algorithms by in vivo RCM in different types of skin tumors [21], inspired by the diagnosis algorithms in dermatoscopy, but permanently correlated with histopathology.

In our one–year experience with confocal microscopy, we encountered several situations that clearly show the importance and accuracy of this matter in pre–surgical diagnosis.

All these examples from our daily practice clearly show that the confocal microscopy, therefore optical biopsy, can guide the clinician towards an accurate diagnosis before surgical removal.

In the end, on case–to–case basis, it all comes down to the examiner's gathered experience, and to his/her decision if, surgical removal is needed or not. Therefore, in certain cases, unnecessary biopsies and surgery can be avoided [22].

In the framework of the present paper, we aimed to establish the rank of RCM in the complex algorithm of skin cancer diagnosing. Moreover, the presented experience can open new possibilities to implement this automated image analyzing the system in practice. The development of this technique can improve the potential of future teledermatologic applications.

Acknowledgement. This paper is partially supported by the Sectoral Operational Programme Human Resources Development, financed from the European Social Fund and by the Romanian Government under the contract number POSDRU/89/1.5/S/64153. The VivaScope 1500 equipment (Lucid Inc, Rochester, NY) and Fotofinder Dermoscope II   was  purchased in the frame of 26/CP/I/2007 Project. Authors would like to thank student Irina Radu for language technical assistance.
